# Deep Learning Enables Instant and Versatile Estimation of Rice Yield Using Ground-Based RGB Images

**DOI:** 10.34133/plantphenomics.0073

**Published:** 2023-07-28

**Authors:** Yu Tanaka, Tomoya Watanabe, Keisuke Katsura, Yasuhiro Tsujimoto, Toshiyuki Takai, Takashi Sonam Tashi Tanaka, Kensuke Kawamura, Hiroki Saito, Koki Homma, Salifou Goube Mairoua, Kokou Ahouanton, Ali Ibrahim, Kalimuthu Senthilkumar, Vimal Kumar Semwal, Eduardo Jose Graterol Matute, Edgar Corredor, Raafat El-Namaky, Norvie Manigbas, Eduardo Jimmy P. Quilang, Yu Iwahashi, Kota Nakajima, Eisuke Takeuchi, Kazuki Saito

**Affiliations:** ^1^ Graduate School of Agriculture, Kyoto University, Kitashirakawa Oiwake-chou, Sakyo-ku, Kyoto 606-8502, Japan.; ^2^ Graduate School of Environmental, Life, Natural Science and Technology, Okayama University, 1-1-1, Tsushima Naka, Okayama 700-8530, Japan.; ^3^ Graduate School of Mathematics, Kyushu University, 744, Motooka, Fukuoka Shi Nishi Ku, Fukuoka 819-0395, Japan.; ^4^ Graduate School of Agriculture, Tokyo University of Agriculture and Technology, 3-5-8 Saiwaicho, Fuchu, Tokyo 183-8509, Japan.; ^5^ Japan International Research Center for Agricultural Sciences, 1-1 Ohwashi, Tsukuba, Ibaraki 305-8686, Japan.; ^6^ Faculty of Applied Biological Sciences, Gifu University, 1-1 Yanagido, Gifu 501-1193, Japan.; ^7^ Artificial Intelligence Advanced Research Center, Gifu University, 1-1 Yanagido, Gifu 501-1193, Japan.; ^8^ Tropical Agriculture Research Front, Japan International Research Center for Agricultural Sciences, 1091-1 Maezato, Ishigaki, Okinawa 907-0002, Japan.; ^9^ Graduate School of Agricultural Science, Tohoku University, Aramaki Aza-Aoba, Aoba, Sendai, Miyagi 980-8572, Japan.; ^10^ Africa Rice Center (AfricaRice), 01 BP 2551 Bouaké, Côte d'Ivoire.; ^11^ Africa Rice Center (AfricaRice), Regional Station for the Sahel, B.P. 96, Saint-Louis, Senegal.; ^12^ Africa Rice Center (AfricaRice), P.O. Box 1690, Ampandrianomby, Antananarivo, Madagascar.; ^13^ Africa Rice Center (AfricaRice), Nigeria Station, c/o IITA, PMB 5320, Ibadan, Nigeria.; ^14^ Latin American Fund for Irrigated Rice - The Alliance of Bioversity International and CIAT, Km 17 Recta Cali-Palmira, C.P. 763537, A.A. 6713, Cali, Colombia.; ^15^ Rice Research and Training Center, Field Crops Research Institute, ARC, Giza, Egypt.; ^16^ Philippine Rice Research Institute (PhilRice), Maligaya, Science City of Muñoz, 3119 Nueva Ecija, Philippines.; ^17^ International Rice Research Institute (IRRI), DAPO Box 7777, Metro Manila 1301, Philippines.

## Abstract

Rice (*Oryza sativa* L.) is one of the most important cereals, which provides 20% of the world’s food energy. However, its productivity is poorly assessed especially in the global South. Here, we provide a first study to perform a deep-learning-based approach for instantaneously estimating rice yield using red-green-blue images. During ripening stage and at harvest, over 22,000 digital images were captured vertically downward over the rice canopy from a distance of 0.8 to 0.9 m at 4,820 harvesting plots having the yield of 0.1 to 16.1 t·ha^−1^ across 6 countries in Africa and Japan. A convolutional neural network applied to these data at harvest predicted 68% variation in yield with a relative root mean square error of 0.22. The developed model successfully detected genotypic difference and impact of agronomic interventions on yield in the independent dataset. The model also demonstrated robustness against the images acquired at different shooting angles up to 30° from right angle, diverse light environments, and shooting date during late ripening stage. Even when the resolution of images was reduced (from 0.2 to 3.2 cm·pixel^−1^ of ground sampling distance), the model could predict 57% variation in yield, implying that this approach can be scaled by the use of unmanned aerial vehicles. Our work offers low-cost, hands-on, and rapid approach for high-throughput phenotyping and can lead to impact assessment of productivity-enhancing interventions, detection of fields where these are needed to sustainably increase crop production, and yield forecast at several weeks before harvesting.

## Infographic

**Figure:**
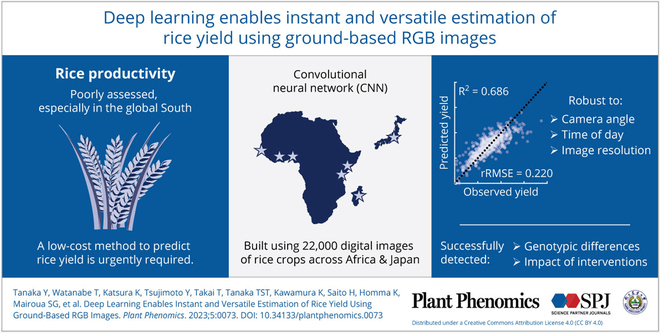

## Introduction

The global demand for staple crop products is expected to increase by 60% by 2050 because of the increased population, per capita income growth, and use of biofuels [1]. As the conversion of carbon-rich and natural ecosystems to cropland exacerbates climate change and biodiversity loss, sustainable intensification of the existing cropland is needed to meet this estimated future demand by reducing yield gap and negative environmental impacts [2,3]. Despite the importance of these goals, crop productivity is poorly assessed, especially in the global South, where there is need to monitor agricultural productivity and evaluate the impact of productivity-enhancing interventions [4]. There are 3 well-known approaches for assessing crop yield, which include self-reporting, crop cutting, and remote sensing. However, self-reported data from smallholder farmers are often inaccurate [5]. Crop cut, wherein a subsection of a plot is physically harvested, is time- and labor-consuming and difficult to scale to large areas with financial limitations. Remote sensing technologies such as satellites and unmanned aerial vehicles (UAVs) with specialized sensors have the capability to assess the crop productivity at scale, but they have not been fully utilized especially in the global South. The absence of reliable data on agriculture statistics is a serious constraint for both agricultural research and policy.

With recent advancement in computational technology, ground-based images captured by low-cost devices together with so called “machine learning” approaches have received great interest. Machine learning technology is one of the most remarkable innovations in the last decade [6,7]. Deep learning is categorized as supervised machine learning and mainly consists of convolutional neural network (CNN). A remarkable feature of CNN is its capability for image analysis. It has already been applied in various situations, which include language translation [8], protein structure prediction [9], board games [10], and agriculture [11,12]. Developing a practical CNN model requires a large-scale combination of images and supervising data. The desirable target objects or crop characteristics could be those that are relatively easy to be visually evaluated for massive data collection. For these reasons, many earlier studies applying CNNs to agriculture focused on the classification of crop biotic [13–15] and abiotic stresses [16], and estimation of crop-growth-related traits such as biomass [17–20], leaf area index [21], grain number [22], and panicle density [23,24]. Recently, some studies demonstrated the direct estimation of crop growth status including yield in specific growth environments and cultivars [25,26]. However, to the best of our knowledge, no study has achieved the versatile estimation of crop yield covering wide range of genotypic and environmental diversity based on CNNs.

This study focuses on rice, which is by far the most important among the big 3 cereals in terms of human consumption in low- and lower-middle income countries and is mainly cultivated by smallholder farmers [27]. We established a database of ground-based digital images of rice taken during the ripening stage and at harvest, and the corresponding yields were collected from 7 countries using a standardized data collection procedure. We then developed a CNN model that covered a wide range of yield levels, rice growing environments, cultivars, and crop management practices, such as crop establishment methods and fertilizer management. We assessed the robustness of the model under various conditions that potentially affected the yield estimation. We demonstrate that rice yield can be rapidly and effectively estimated at a low cost in diverse light environments at harvest and during the late ripening stage, without labor-intensive crop cuts or knowledge-intensive remote sensing technologies.

## Materials and Methods

### Construction of database for rice canopy image and rough grain yield

**Fig. 1.**
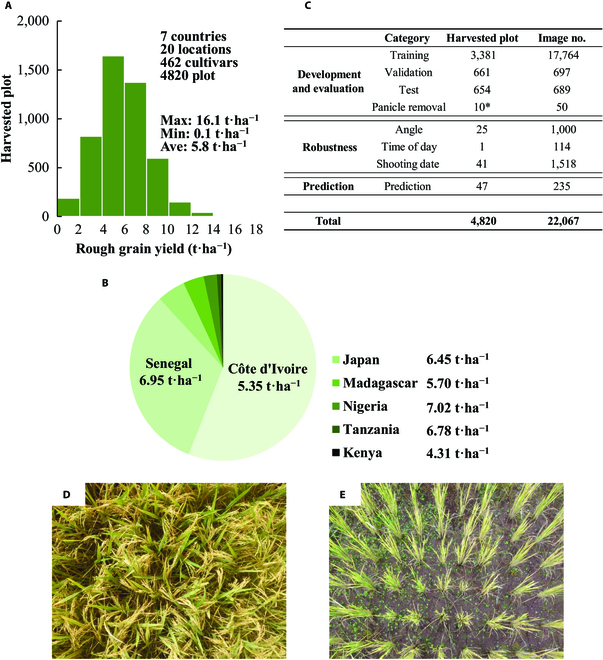
The global database of the rice canopy image and corresponding rough grain yield. (A) Bar plot depicting the frequency distribution of observed rough grain yield in the database collected at 7 countries. (B) Pie chart of the dataset composition of 7 countries with the average rough grain yield in each country. (C) Tabular representation of the dataset classified into 3 major parts: development and evaluation, robustness, and prediction. For the subcategories, the training and validation datasets are directly used to develop the model. The test and panicle removal datasets were used to evaluated the accuracy and characteristics of the model. For details of angle, time of day, and shooting date, please refer to the main text. The prediction dataset consists of the data at Tanzania and Japan (Tokyo University of Agriculture and Technology), which are not included to any other categories. Asterisk indicates that 10 different yield data were generated for single plot of cv. Koshihikari at Kyoto, Japan by the sequential panicle removal. (D and E) Images of the rice canopy showing the highest (D) and the lowest (E) rough grain yield, which is collected in Senegal and Madagascar, respectively.

Field campaigns were conducted in 2019 and 2020 at 20 locations in 7 countries (Côte d'Ivoire, Senegal, Japan, Kenya, Madagascar, Nigeria, and Tanzania). Data on rice growth traits and digital images were collected in seed production plots and experimental fields at research stations and farmers’ fields (Table S1). At maturity, the red-green-blue (RGB) images were captured vertically downward over the rice canopy from a distance of 0.8 to 0.9 m using a digital camera (Fig. S1A). The camera was set to automatic mode. The focal length and aspect ratio were set to 28 mm and 4:3 or 16:9, respectively. All the images were saved as jpg files. The digital cameras used in this study are listed in Table S1. The rice canopy images covered 1 m^2^, which correspond to the harvesting area proposed by Food and Agriculture Organization and used by Japan for agricultural statistics [28]. Rough grain yield that contained filled and unfilled grains was measured at the corresponding plot or larger plots, where yield data were collected on the basis of field experiments (Table S1). Rice yields were reported as 14% moisture content. The aboveground total dry weight and filled grain weight were also recorded in most studies, but not used for the CNN-based estimation because of the lack of data in some cases. Rice yield level, rice production system, rice cultivar, and key crop management practices are shown in Table S1. The database consists of 8 categories, as presented in Fig. 1C. For most of the training, validation, and test data, a single image per plot was recorded. These 3 categories are the main part of the database and randomly split by a ratio of 5:1:1. After splitting the data, the images categorized in the training data were augmented for 4-fold by flipping horizontally, vertically, and their combination, which resulted in 17,764 images for training data. For panicle removal, angle, shooting date (see the following sections), and prediction data, we used 5 replicated images per plot. These 5 images were recorded by swaying the camera by 1 to 2 cm horizontally. The prediction data consisted of the dataset collected at Moshi (3.45S, 37.38E), Tanzania, and at Tokyo (35.41N, 139.29E), Japan, where the data were not included in any other categories. For the time-of-day data, the sequential shooting of the canopy images was conducted using a fixed camera. In total, 4,820 yield data and 22,067 images of 462 rice cultivars were used in this study (Fig. 1C and Table S2).

### Panicle removal and experiments for robustness evaluation

The panicle removal experiment was conducted at Kyoto (35.2N, 135.47E) and Tsukuba (36.03N, 140.04E), Japan. The 5 replicated canopy images were acquired for the plot to be harvested. Two panicles per hill at the random position of the canopy were removed, and then 5 images were acquired. The grain weight from the collected panicles was measured separately. By repeating this process until all the panicles were removed from the harvesting plot, the series of images with gradually decreased panicle number and the corresponding yield were obtained. The dataset at Tsukuba was included for the training, validation, and test data, and the dataset at Kyoto was used to evaluate the impact of canopy removal on the yield estimation.

The angle changing experiment was conducted at M’bé (7.87N, 5.11W), Côte d'Ivoire. The curved rail with a diameter of 1.8 m was fixed above the canopy to be harvested. By shifting the position of the camera on the rail, the image from the various depression angles were shot with the constant center of the image. The depression angles were set to 20°, 30°, 40°, 50°, 60°, 70°, 80°, and 90° (control). The data for angle changing experiment were collected for 25 harvested plots. The day time experiment was conducted at Kyoto, Japan. HykeCam SP2 (Hyke Inc., Japan) was fixed above the canopy of cv. Koshihikari and Takanari. The canopy images were automatically recorded every 30 min 5 days before the date of harvest for Koshihikari and 11 days before harvest for Takanari. After finishing the record, the plot was harvested by the common protocol with other experiments. The data of Takanari were used for the model development, and the data of Koshihikari were used for the time-of-day analysis.

The shooting date experiment was conducted at M’bé, Côte d'Ivoire and Marovoay, Madagascar. At M’bé, the 22 cultivars grown in 34 plots in total were used. The canopy images of these plots were acquired once a week from 1 to 4 weeks after 50% heading (WAH), 2 days and 1 day before harvest, and at harvest. Only the images taken on 2 days and 1 day before harvest were used for model development, while the others were used for the shooting date analysis. After the final image records, the rice plants were harvested using a common protocol. At Marovoay, the canopy images of 7 plots were recorded from 2 days before 14 days after 50% heading. Six images were taken every 10 min from 1200 to 1250 h and were used for the shooting date analysis.

### Image processing and development of CNN model

The RGB images of the rice canopy were recorded with an aspect ratio of 4:3 or 16:9. For the images recorded at 16:9, the edge of the long side was trimmed to a ratio of 4:3. The images were then resized to 600 × 450 pixels for recording in the database. A bilinear algorithm was used to resize the images. This resize was to eliminate the difference in pixel sizes of images from various cameras, while keeping the aspect ratio and ground sampling distance (GSD). The images used for the analyses have the GSD of 0.2 cm·pixel^−1^. The images were again resized to a square of 512 × 512 pixels in 8-bit PNG format as inputs for the CNN model. The brightness values of each channel of RGB were divided by 255 to scale from 0 to 1. These values were then standardized using the mean and variance calculated from all images categorized in the training dataset. The mean and variance of the RGB channel for the training dataset were [R, G, B] = [0.490, 0.488, 0.281] and [0.230, 0.232, 0.182], respectively. The structure of the CNN was developed by Neural Network Console software version 1.5 (Sony Network Communications Inc., Japan, https://dl.sony.com/). The Neural Network Console is a graphical user interface (GUI)-based software for Windows OS to design the structure of CNN and perform the training of the model. The database of the RGB images and rough grain yield was imported to the Neural Network Console, and the optimal structure showing the lowest validation error was determined by the CNN structural search function of the software. During the structural search, the loss function and optimizer were defined by the mean absolute error and Adam optimizer, respectively. The batch size, learning rate, and epoch number were set to 32, 0.001, and 50, respectively. The determined CNN structure (Fig. S2), loss function, and optimizer were then deployed using Python language (version 3.7) with PyTorch framework (version 1.7). The optimal learning rate and batch size were determined by changing the combination of these hyperparameters. Batch sizes of 16, 32, 64, and 128, and learning rates of 0.0001, 0.0002, 0.0005, 0.0008, and 0.001 were combined, and the learning process was replicated 10 times for each combination. The epoch number was set to 100, and the learning process was conducted by minimizing the loss of estimated and observed yields in the training dataset. The validation loss was also calculated for every epoch, and the model showing the least loss for validation was recorded. The relative root mean square error (rRMSE) for the test dataset was calculated for models with all combinations of the hyperparameters and averaged across 10 replications. The best combination of batch size and learning rate was determined, and the recorded model was used in the present study.

To evaluate the model accuracy with the images of lower resolutions, we additionally developed the sets of training, validation, and test images with GSD of 0.4, 0.8, 1.6, and 3.2 cm·pixel^−1^. The CNN models were trained using images having these lower resolutions. The framework, optimizer, and the epoch number were identical with the establishment of the default model. On the basis of the optimization for the default model, the batch size and learning rate were set to 32 and 0.0001, respectively. The learning process was replicated 5 times for each GSD condition. The validation loss was also calculated for every epoch, and the model showing the least loss for validation was recorded. The *R*^2^ value for validation and test dataset was calculated for each selected model and averaged across 5 replications. The altitude of the UAV and the single image footprint that gives the specific GSD was calculated by assuming the camera spec with the focal length of 10 mm, an image sensor size of 1 inch (2.54 cm), and a pixel size of 20 megapixels.

### Occlusion-based method to quantify the additive effect on the yield estimation

The occlusion-based method [29] was applied to visualize the spatial distribution of the additive effect on yield estimation. The image of the rice canopy with 450 × 600 pixels was partly masked by the gray square with a brightness of [R, G, B] = [0.5, 0.5, 0.5]. The size of the gray square was 30 × 30 pixels. By shifting the position of the gray square by 30 pixels for both the row and column directions of the image array, 300 images were generated per original image (Fig. S5A and B). Each portion of the original image was covered by one of the images in a series of 300 images with a gray square. Then, the rough grain yield was estimated using the CNN model, and the subtraction against the estimation for the original image was calculated. These values overlapped with the original image as a heatmap (Fig. S5C).

### Statistical analyses, data summarizing, and code availability

The 4,820 observations of rough grain yield data were summarized by calculating the average, maximum, and minimum yields. The data were categorized according to the collected country, and the average yield in each country was calculated. The *R*^2^ and rRMSE were calculated to evaluate the model performance in each analysis. The rRMSE is defined as follows:

$$\frac{1}{\bar{y}}\sqrt{\frac{1}{n}\sum_{k=1}^{n}{\left(f_{i}-y_{i}\right)}^{2}}$$ (1)

where $\bar{y}$ is the average of the observed yield, *n* is the size of the data, and *f_i_* and *y_i_* are the individual estimations and observations of the yield. The *t* test and 2-way analysis of variance (ANOVA) were conducted for the prediction dataset collected in Japan and Tanzania, respectively. The rough grain yield for panicle removal, angle, shooting date, and prediction dataset was estimated with 5 replicated images per harvested plot and then averaged. The standard error of the 5 replicated estimations was calculated in the panicle removal experiment. For the changing angle experiment, the first, second, and third quartiles were calculated for the deviation between the estimated and observed yields across 25 plots and displayed with their average, maximum, and minimum values as the box plot. For the day time experiment, the estimated yield for every 30 min was averaged across successive 6 days, and the standard error was calculated. Segmented linear regression was adopted to determine the relationship between days after 50% heading and the relative yield observed in the shooting date experiment. The data collected at M’bé, Côte d'Ivoire:

$$y=a+\mathrm{bx}+b\left(c_{1}-x\right)∙I_{\left(x-c_{1}\right)}$$ (2)

and for the data collected at Marovoay, Madagascar:

$$y=a+b\left(x-c_{1}\right)∙I_{\left(x-c_{1}\right)}+b\left(c_{2}-x\right)∙I_{\left(x-c_{2}\right)}$$ (3)

were used, respectively. The parameters *a* and *b* are constant, *y* is the ratio between the observed and the final yield, and *x* is the date after 50% heading. The parameters *c*_1_ and *c*_2_ are the breaking points of the segments, and Eq. 3 represents the 3 segmented regressions. Function ‘*I*’ is the step function, which is defined as follows:

Ix=0,x≤01,x>0 (4)

For the dataset in Madagascar for the shooting date experiment, the 6 estimations from 1200 to 1250 h were averaged and defined as an estimation for a plot. The estimations at 7 harvested plots were then averaged, and the standard error was calculated. All analyses in the present study were conducted using Microsoft Excel (Microsoft, Redmond, WA, USA), Neural Network Console software (Sony Network Communications Inc., Japan), and Python language version 3.7 (http://www.python.org) with PyTorch framework version 1.7 (https://pytorch.org/). The code to run the developed CNN model is available at https://github.com/r1wtn/rice_yield_CNN.

## Results

### Database on rice canopy image and grain yield

The multinational dataset of rice canopy image and corresponding rough and filled grain yields and aboveground dry weight was established with a standardized data collection procedure for 4,820 harvested plots and 22,067 images across 20 locations in 7 countries (Fig. 1A, Fig. S1, and Tables S1 and S3). Côte d'Ivoire, Senegal, and Japan accounted for 56%, 32%, and 5% of total plots, respectively (Fig. 1B). The dataset covers both lowland and upland rice production systems containing 462 rice cultivars and includes 2 crop establishment methods (direct seeding and transplanting) (Table S2). N-P-K fertilizer application ranged from 0 to 200 kg of N·ha^−1^, 0 to 120 kg of P_2_O_5_·ha^−1^, and 0 to 120 kg of K_2_O·ha^−1^, respectively (Table S1). The observed rough grain yield ranged from 0.1 to 16.1 t·ha^−1^ with an average of 5.8 t·ha^−1^ and showed a normal distribution (Fig. 1A). As rough and filled grain yields and aboveground dry weight were highly correlated each other (Fig. S3), further data analyses using the CNN model focused only on rough grain yield.

### A CNN model to estimate rough grain yield from canopy image

**Fig. 2.**
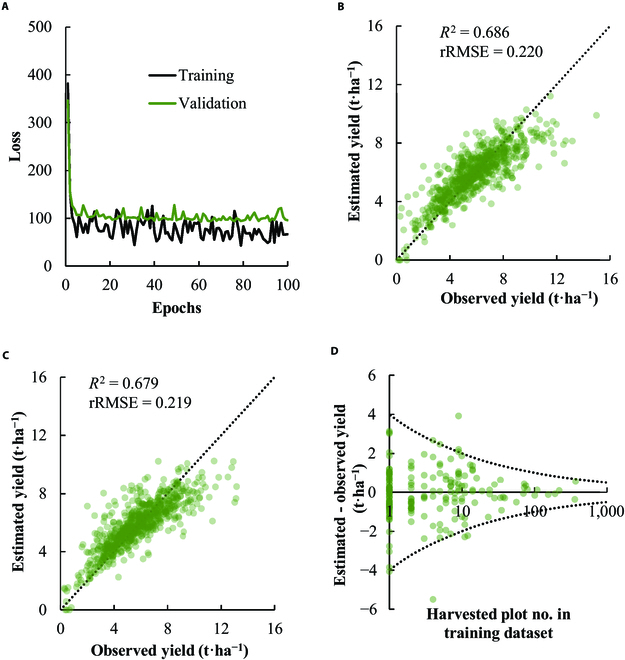
Development of the deep-learning-based model to estimate the rough grain yield of rice. (A) Graph illustrating the learning curve for the training and validation dataset. The minimum loss for the validation dataset was recorded at an epoch of 61, and the model was used for all the analyses in the present study. (B and C) Scatter plots depicting the estimation of the rough grain yield in the validation (B) and the test (C) datasets. The dotted line represents the 1:1 relationship. (D) Scatter plot showing the difference between estimated and observed yield of each cultivar in test dataset plotted against the number of harvested plots in training dataset. The dotted line represents $y=4\cdot {\left(\frac{1}{2}\right)}^{{\text{log}}_{10}x}$.

The determined CNN structure had 5 convolutional layers in the main stream and the 4 convolutional layers in the branching stream (Fig. S2). The pooling layers included both of Average Pooling and Max Pooling. The rectified linear unit (ReLU) was mainly chosen as the activation function, but the exponential linear unit (ELU) and LeakyReLU were also used in some parts. In the head part of CNN, the information from the 2 streams were fully connected, followed by the last ReLU layer to output the estimated yield. The total number of parameters of the structure was 41,017. The learning rate and batch size during the learning process were optimized with 10 replications and identified the best combination at 0.0001 and 32, respectively, for the test dataset (Fig. S4). With this combination, the best model of the learning process was generated at an epoch of 61, and the model was used for all of the following analyses (Fig. 2A) , except for the test of greater GSD images. The developed CNN model could explain 69% and 68% of the variation in yield for validation and test data, respectively, with an rRMSE of 0.22 for both (Fig. 2B and C). The relationship between the observed and estimated yields fit well to the 1:1 line for both datasets. The deviation between the estimated and observed yields of individual cultivars in the test dataset was plotted against the number of harvested plots in the training dataset (Fig. 2D). The cultivars with more than 25 harvesting plot in the training dataset tended to have less than 1.5 t·ha^−1^ deviation. The empirical relationships illustrated as upper and lower boundary curves in Fig. 2D indicate that increasing the number of plots by 10 times can reduce the error of the yield estimation by 50%.

**Fig. 3.**
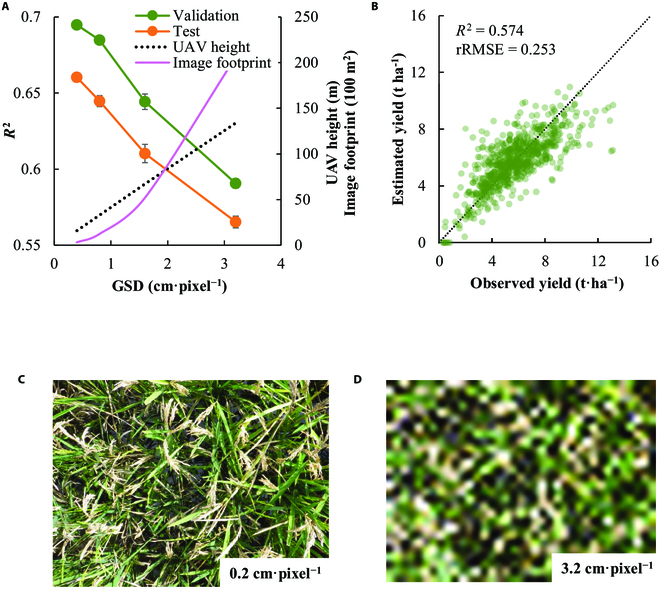
The accuracy of the yield estimation by the models established using various resolutions of the images. (A) The relationship between *R*^2^ values of the models and GSD in the validation and test dataset. The dotted line represents the corresponding height of the camera when assuming the UAV with a focal length of 10 mm, an image sensor size of 1 inch, and a pixel size of 20 M. (B) Scatter plots depicting the estimation of the rough grain yield in the test dataset with images of 3.2 cm·pixel^−1^. (C and D) An example of the image with GSD of 0.2 cm·pixel^−1^ (original) and 3.2 cm·pixel^−1^, respectively.

The applicability of the CNN to the images with greater GSD was then evaluated by comparing the models developed by the various resolutions of the image dataset. Compared with the default model (GSD = 0.2 cm·pixel^−1^), the model based on the greater GSD showed the lower accuracy both with the validation and test dataset (Fig. 3A). The *R*^2^ value for the test dataset was, however, greater than 0.55 even when the model was trained by the images with a GSD of 3.2 cm·pixel^−1^ (Fig. 3B). When assuming the typical camera specs of UAV, this GSD corresponds with altitude of 134 m and single image footprint of 2.06 ha (Fig. 3A).

**Fig. 4.**
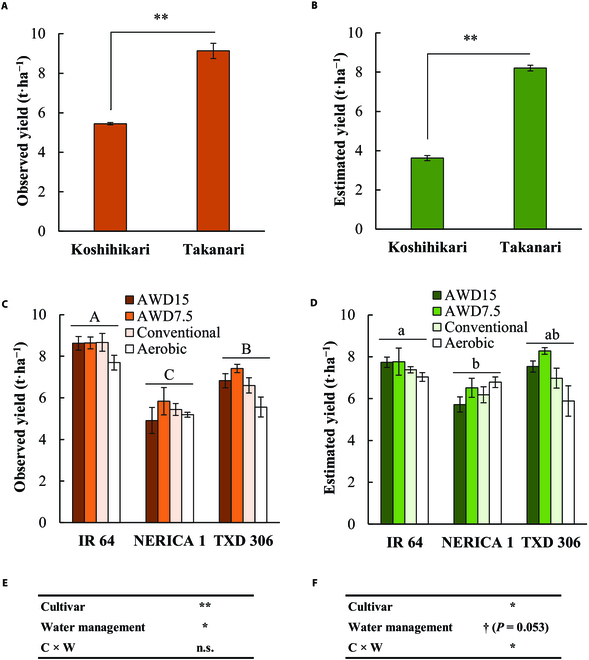
The prediction of the rough grain yield of datasets independent from the model establishment. (A and B) Comparison of observed (A) and estimated (B) rough grain yield of Koshihikari and Takanari harvested in Tokyo, Japan. Bars indicate the standard error (*n* = 5 for Koshihikari and *n* = 6 for Takanari). (C and D) Comparison of observed (C) and estimated (D) rough grain yield of IR 64, NERICA1, and TXD 306 with different water managements in Tanzania. AWD15 and AWD7.5 represent the intermittent irrigation up to 5 cm in depth when water level decreases under 15 and 7.5 cm of soil surface, respectively. Conventional represents the fully irrigated condition, and aerobic represents the upland condition with saturated ground water. The same alphabets upon the horizontal line above the bars represents the no significance at *P* < 0.05 (Tukey’s test) when averaged across water managements. (E and F) The results of 2-way ANOVA for observed (C) and estimated (D) rough grain yield in Tanzania experiment. C x W represents the interaction of cultivar and water management. ^**^P < 0.01, ^*^P < 0.05, and ^†^*P* < 0.1. n.s., not significant.

The accuracy of the default CNN model was further evaluated using the prediction dataset. The observed yield of cv. Takanari was significantly greater than that of cv. Koshihikari (*P* < 0.01; Fig. 4A) in Tokyo, Japan. The CNN model successfully detected this cultivar difference in yield (*P* < 0.01; Fig. 4B). In Tanzania, 4 different water managements were applied to 3 cultivars including cv. TXD 306, which was included solely in the prediction dataset. There were significant main effects of cultivar and water management on observed yield (Fig. 4C and E). The CNN model detected the cultivar difference across water management practices (Fig. 4D and F). There was significant difference in both observed and estimated yield between IR 64 and NERICA 1. While difference in observed yield between TXD 306 and others was significant, this was not the case for estimated yield. The model slightly failed to reach eightstatistical significance for water management (*P* = 0.053; Fig. 4F). This is due to the overestimation in aerobic treatment in NERICA 1, but the yield variation associated with water management practices was successfully predicted for other cultivars.

To understand how the CNN model reads the images and estimates rice yield, we used the occlusion-based visualization technique to estimate the additive effect on yield estimation [27]. Briefly, the specific part of the image was masked by a gray square, and the yield estimation of the masked image was subtracted from that of the original image. The calculated values can be interpreted as the additive effect of the masked region on the yield estimation and mapped to the original image with a color scale (Fig. S5). This analysis revealed that the regions containing many rice panicles have a positive effect, whereas the region with leaves, stems, or ground has a negative effect on yield estimation.

**Fig. 5.**
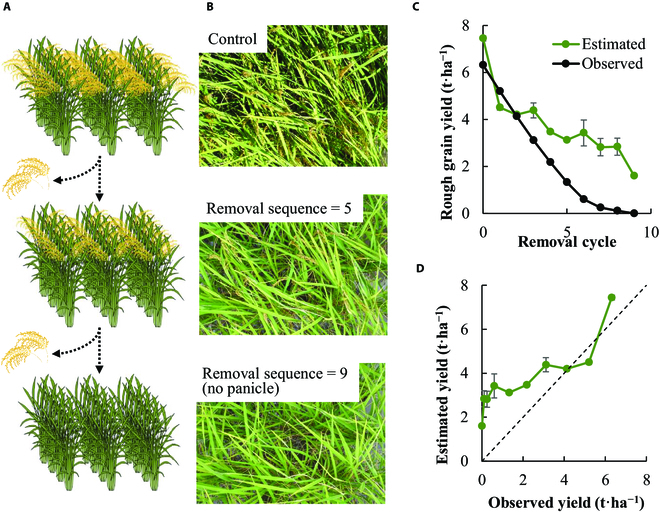
Panicle removal experiment to visualize the quantitative contribution of the panicles to the yield estimation. (A) Schematic illustration of the panicle removal. The image was taken at the fixed camera above the rice canopy (cv. Koshihikari). Two panicles per plant were removed, and the image was taken 5 times with the random shift. This cycle was repeated until all the panicles were removed from the canopy. (B) The canopy image before panicle removal, removal cycle = 5 and 9 (no panicle). (C) Line graph of the observed and the estimated yield plotted against the removal cycle. (D) Scatter plot of observed and estimated yield for the canopies generated by the panicle removal. The bars represent the standard error (*n* = 5).

The importance of panicles for yield estimation was further validated using panicle removal experiment in Kyoto, Japan. Two panicles per hill were sequentially removed from the canopy, and the rough grain weight and canopy images were recorded for each sequence (Fig. 5A and B). The yield was estimated using the CNN model for each sequence of panicle removal. As more panicles were removed, the estimated yield were gradually reduced (Fig. 5C and D). When all panicles were removed, the CNN model predicted yield of 1.60 t·ha^−1^.

### Robustness of the developed CNN model

The robustness of the CNN model to image quality was tested using the images taken (a) from different shooting angles, (b) at various times of day during the 5 days before harvest, and (c) on different shooting dates during the ripening stage. The shooting angle assumes human error, while the time of day reflects the changing natural environment causing the variation of the contrast or color balance of the image. The shooting date is important to assess when rice yield can be effectively predicted using our model during the ripening stage.

**Fig. 6.**
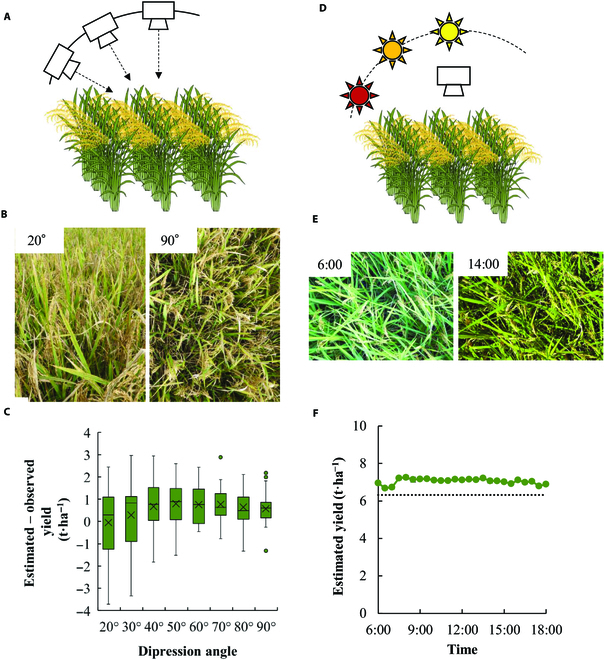
The effect of the depression angle and time of the day on the yield estimation. (A) Schematic illustration of the protocol to take the canopy image with various depression angles. The digital camera was sided on the rail above the rice canopy to change the depression angle with the same rice canopy. The image at each angle was taken 5 times with the random shift. (B) The examples of the canopy image taken from 20° and 90° of depression angle. (C) Boxplot of the relative error of the estimated to the observed yield against the depression angle of the image. (*n* = 25). (C) Schematic illustration of the protocol to take the canopy image with various shooting times of the day. The camera was fixed above the rice canopy (cv. Koshihikari), 5 days before harvesting, and the image was collected until the harvest day. The image was taken every 30 min from 0600 to 1800 h. (E) The examples of the canopy image taken at 0600 and 1400 h. (F) Plot of the estimated rough grain yield against the time of day. The data of 6 days were averaged, and the standard error is represented by bars (*n* = 5). The dotted line represents the observed rough grain yield of 6.31 t·ha^−1^.

To determine the range of shooting angles acceptable for the developed CNN model, we estimated rice yield using images acquired from 8 shooting angles [in 10° increments from 20° to 90° (control)] in M’bé, Côte d'Ivoire (Fig. 6A and B). The deviation between the estimated and observed yields was averaged across 25 harvested plots at each angle. The deviation ranged from −3.7 to 2.4 t·ha^−1^ when the depression angle was 20° (Fig. 6C). The deviation decreased with an increase in the depression angle. When the outlier was excluded, the ranges of the deviation were between −0.45 and 2.44 t·ha^−1^ at 60°, which was comparable with that at 90° (control). The estimation accuracy analysis showed that greater depression angles resulted in better estimation accuracy (Fig. S6). When the depression angle was greater than 60°, the *R*^2^ and rRMSE calculated between the estimated and observed yields ranged from 0.435 to 0.493 and 0.180 to 0.219, respectively.

The image of the rice canopy was captured by a fixed-point camera every 30 min for 5 successive days before and at harvest in Kyoto, Japan (Fig. 6D and Fig. S7). The images at 0600 and 1400 h on 29 August 2020, having a different color balance and contrast, are shown as an example of a clear sunny day (Fig. 6E). Despite such variations in light environments, the CNN model provided stable outputs throughout the daytime with a slight overestimation (Fig. 6F).

**Fig. 7.**
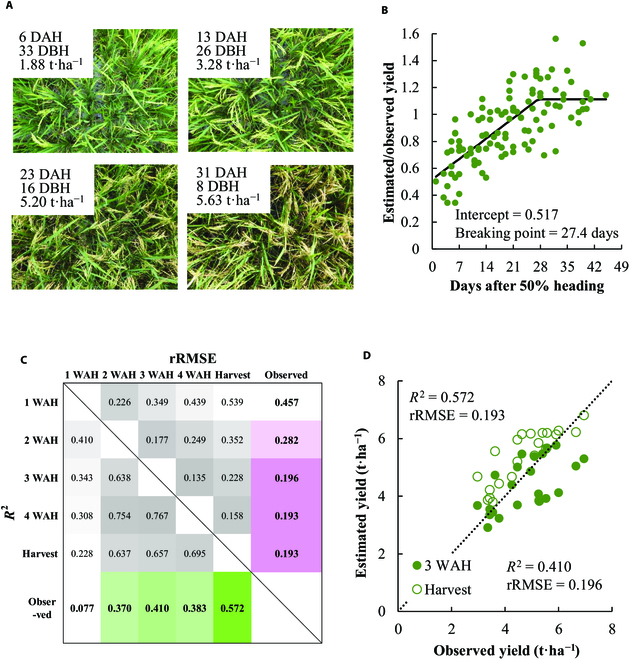
The applicability of the model to the images taken during grain filling stage. (A) Examples of the image taken at approximately 1 to 4 WAH (cv. IRRI 154). (B) Scatter plot of the estimated yield relative to the final yield plotted against days after 50% heading. The data consist of images of 22 cultivars taken at 1 to 4 WAH. The solid line represents the segmented linear regression. (C) The matrix of rRMSE and *R*^2^ with the observed and estimated yield for images taken at approximately 1 to 4 WAH and at harvest. (D) Scatter plot depicting the relationship between observed and estimated yield at 3 WAH and at harvest. The dotted line represents the 1:1 relationship.

To assess from when the CNN model can predict rice yield during ripening stage, we took the canopy image once a week after 50% heading until the harvest for 22 cultivars in M’bé, Côte d'Ivoire. The yield estimated in the early ripening stage tended to be lower than the observed yield at harvest, whereas such a trend was not observed with the yield estimated at the later ripening stage (Fig. 7A). This indicates that the model recognizes mature panicles (Fig. 5 and Fig. S5) but not the immature panicles. When the data from 22 cultivars were pooled, the ratio of the estimated yield to the observed yield ranged from 0.3 to 0.6 at just after 50% heading, and the *y* intercept of the segmented regression was 0.517. The ratio increased linearly during ripening. The relationship reached a plateau at approximately 4 WAH (Fig. 7B). A similar trend was also observed in Madagascar (Fig. S8), while the relative yield plateaued within 2 WAH. In M’bé, Côte d'Ivoire, the *R*^2^ between the estimated yield during 2 to 4 WAH and the observed yield ranged from 0.370 to 0.410 and was lower than that between the estimated yield at harvest and observed yield (Fig. 7C). The rRMSE between the estimated yield after 3 WAH and the observed yield ranged from 0.193 to 0.196 and was similar to that between the estimated yield at harvest and the observed yield.

## Discussion

This is the first study to develop a versatile CNN model to predict rice yield accurately only by using ground-based RGB images. In the previous attempts [25,26], the application of the CNN model was tested in the specific growing environments and cultivars. Our model was able to estimate rice yield with satisfactory precision in the existing most comprehensive and international dataset in terms of the growing environments, management practices, number of cultivars, camera angles, and time of days . The accuracy of estimation in the test dataset was comparable to or even higher than those shown in earlier studies that used satellite data, or in combination with other data and models, or in UAVs equipped with various sensors for estimating crop-growth-related traits such as aboveground biomass and leaf area index, or in indirectly predicting crop yield in farmers’ fields [26,30–36]. Furthermore, in this study, the accuracy was evaluated using an independent prediction dataset, which was rarely tested in the earlier studies. In the prediction dataset, the CNN model successfully detected the cultivar difference in yield in 2 locations. However, the model could not detect yield difference between cv. TXD 306 and other 2 cultivars. This might be because TXD 306 was not included in the training and validation dataset. As shown in Fig. 2, increased data for given cultivar in the training dataset can result in improved model accuracy. In addition, our model detected yield difference among water management treatments except for one cultivar (cv. NERICA 1) in Tanzania. The reason for the exception in NERICA 1 is unknown and requires attention in future study.

Dry-weight-based evaluation of the rough grain yield needs at least 48 to 72 h oven-drying [28]. In addition to that, crop cut, threshing, and other processes require additional time and labor inputs. In contrast with this conventional method, the CNN-based estimation is instantaneous, and shooting an image requires a few seconds. Combined with the successful detection of the yield difference among cultivars and water management practices, our model can be applied to the high-throughput phenotyping for on-station agronomic experiments.

Our analyses showed the negative relationship between the model accuracy and GSD of the images used for the model development. This was because the lower resolutions led to the loss of the leaf and panicle architecture (Fig. 3C and D). However, the CNN model trained with the images of GSD of 3.2 cm·pixel^−1^ still shows the sufficient estimation accuracy (Fig. 3A and B). This GSD level is easily achieved by the UAV altitude greater than 100 m, if the commercial RGB camera is used. These results suggest that the CNN model can potentially use the images captured by the UAV for yield estimation. The CNN-based estimation of rice yield and its spatial variation at field level can be a powerful solution for monitoring the rice productivity in the regional scale in the future.

The unknown conditions causing the poor or moderate estimations of the CNN model should always be assumed when considering the scale and diversity of on-farm rice cropping systems globally. For instance, the dataset does not include the canopy affected by severe lodging, pests, insects, weeds, or abiotic stresses such as heat, drought, and flooding. Most of the data are from on-station irrigated lowland rice fields with relatively higher yields, and data from farmers’ fields are limited. Thus, further research should especially focus on low-yielding and rained environments, and assessment of the potential use of the model for stressed or injured rice plants is warranted. The most practical solution to adapt the model to these conditions would be to add these new data to the database and develop a new model. The results in Fig. 2D suggest that better accuracy can be achieved with more harvesting plots, indicating the extensibility of the CNN model. As a criterion, 25 harvesting plots are needed for adaptation to new conditions with practical accuracy (error < 1.5 t·ha^−1^), which should be validated for developing a sampling framework for improving and adapting the model to new conditions.

The object detection algorithm based on CNN enabled to detect panicles of rice [23] and wheat [24] and could offer a potential approach for indirect yield estimation. However, it is well known that other yield components such as number of spikelets per panicle interact with panicles and strongly affect rice yield [37]. Unless the models for predicting other yield components are not developed, the model for detecting panicles would not be useful for the accurate yield estimation. On the basis of the panicle removal experiment (Fig. 5) and the occlusion-based method for visualizing the distribution of the additive effect (Fig. S5), our CNN model also autonomously learned the contribution of panicles to yield only by the relationships between input canopy images and the observed yield. There are several reasons for the overestimated yield of the images after removing panicles. First, apart from the existence of panicles, information about the background canopy may have also been utilized for yield estimation. Second, as images on the immature rice canopy without panicles at harvest were not included in training and validation dataset, the CNN model might not have been able to predict yield well. Third, as our CNN model was developed using plots having relatively high yield, yield prediction at lower yielding conditions could have been less accurate. Although it is difficult to quantify, our model may capture the feature of background canopy, such as the amount of leaves, planting density, or stem size for yield estimation (Fig. 5C and D and Fig. S5). It was also supported by the positive value of the estimation yield for the images taken at around 50% heading date when the panicles were immature (Fig. 7A and B and Fig. S8). Capturing the whole part of the rice canopy may lead to the stable and quantitative estimation of yield because the accumulation of aboveground dry weight is tightly linked to the yield formation (Fig. S3).

The robustness of the CNN model to image quality is crucial because the image is not necessarily acquired under optimal rice growing conditions. On the basis of our assessment of the robustness of the model, the results suggest that (a) the model can be applied to the depression angles of the camera from 60° to 120° (Fig. 6C), (b) the model output is slightly affected by the changing light intensity without any reference board or color checker (Fig. 6F), and (c) the model with images acquired at 3 WAH or later has moderate prediction accuracy for yield forecasting before the harvest (Fig. 7). In particular, yield forecasting has great potential benefits in terms of field management, marketing, distribution, and policy decisions. The yield was estimated earlier without underestimation in Madagascar (i.e., after 2WAH) than Côte d'Ivoire. The reason for such difference between the 2 locations is not known, but it may be combined effect of various factors such as cultivar-specific dynamics of grain filling, growing environment, soil fertility, and water management, and, therefore, further studies are warranted.

The CNN structure used in this study has several convolutional layers (Fig. S2) and is much smaller than the CNN used in the previous study for rice yield estimation [26] or representative structures for image recognition [38]. This implies that the developed model can be easily transferred to mobile devices such as smartphones. The model does not require any type of color checker. It can accept the depression angle of the image from 60° to 120° at any time of the day, at 3 WAH or later, for shooting the canopy image. The model having the sufficient accuracy could be developed with the images of lower resolution, and our approach can be potentially combined with the UAV-based imagery. The present study leads to the high-throughput phenotyping, impact assessment of productivity-enhancing interventions, and identifying fields where these are needed to sustainably increase crop production [39].

## Data Availability

The data that support the findings of this study are available from the authors on reasonable request.
