# Supplementary material for: Deep Learning Enables Instant and Versatile Estimation of Rice Yield Using Ground-Based RGB Images
Source: Plant Phenomics. 2023 Jul 28;5:0073. doi: 10.34133/plantphenomics.0073 (PMC10795498; doi:10.34133/plantphenomics.0073)
Supplement: Supplementary 1 — Tables S1 to S3 Figs. S1 to S8 [file plantphenomics.0073.f1.zip › Supplementary Figures_PP.pdf]

(a)

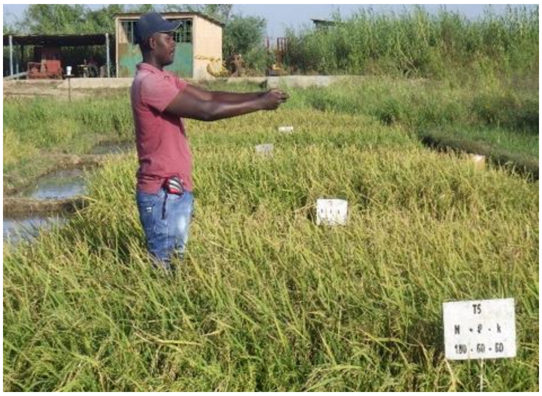

(b)

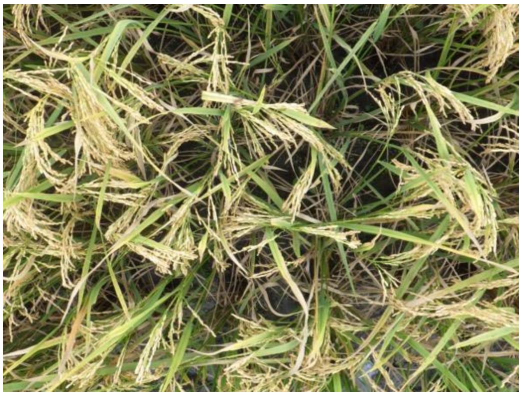

(c)

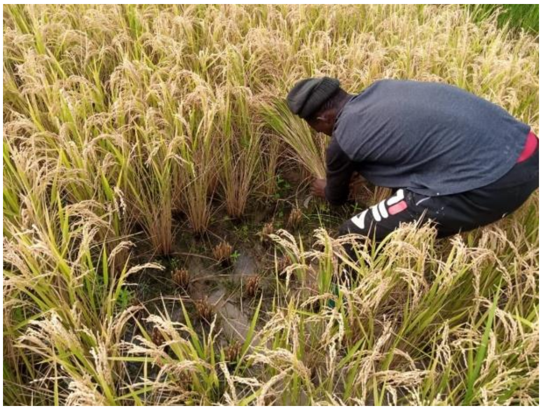

Figure S1. The procedure to take the canopy images and the yield measurement. (a) The image is taken 80-90 cm above the top of rice canopy. The image was taken just before harvesting except for the experiment which is conducted with the various shooting date. The focal length and the aspect ratio was set to 28 mm and 4:3, respectively. (b) Example of the image of cv. IRRI 154. Based on the protocol, the rice canopy of approximately 0.9*1.2 m is covered by the image. (c) The rough grain yield was recorded and the moisture content was set to 14%.

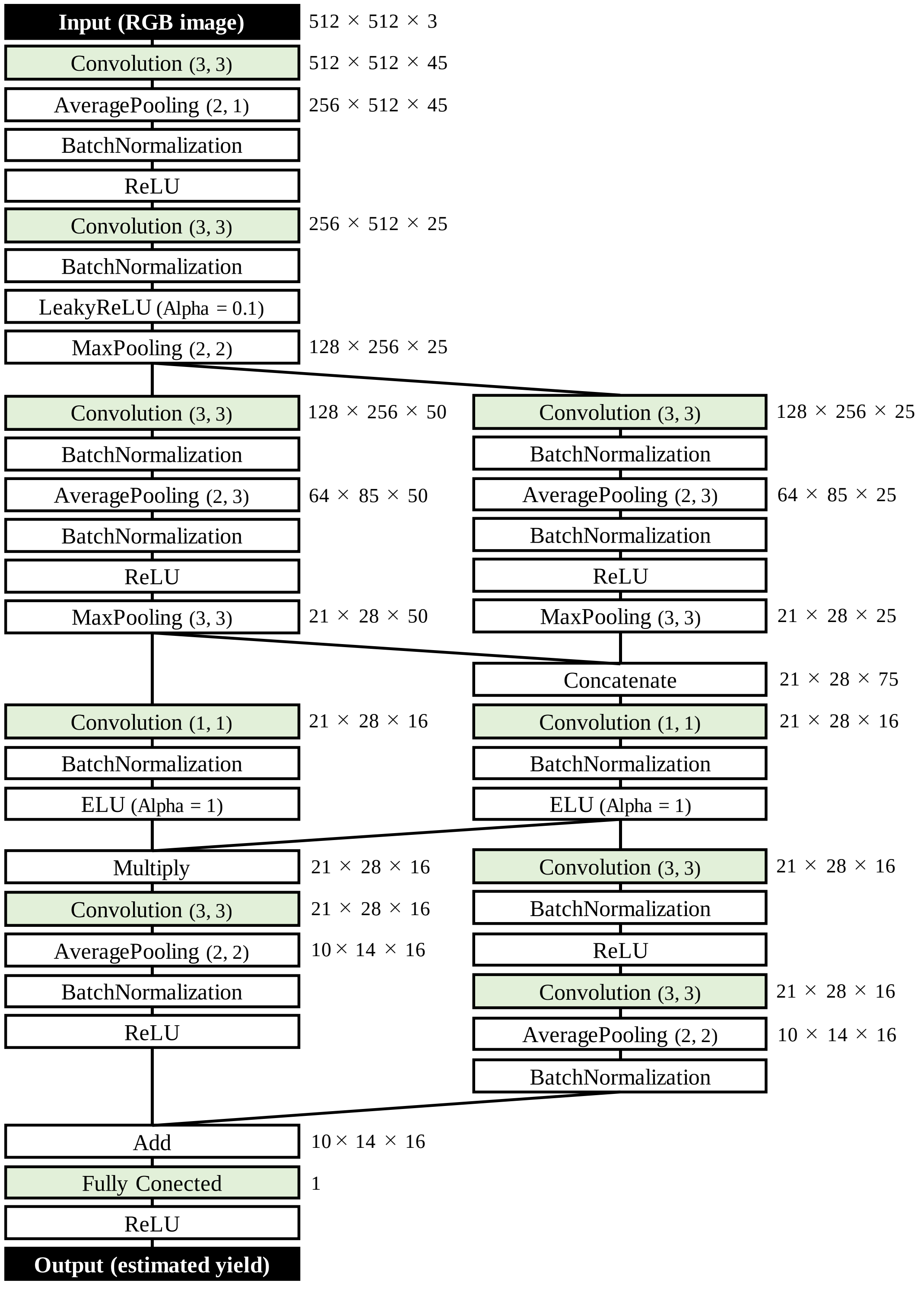

Figure S2. The structure of the CNN model.
The convolutional neural network developed in the present study. The model structure was determined by the structure search function in Neural Network Console (Sony) software.

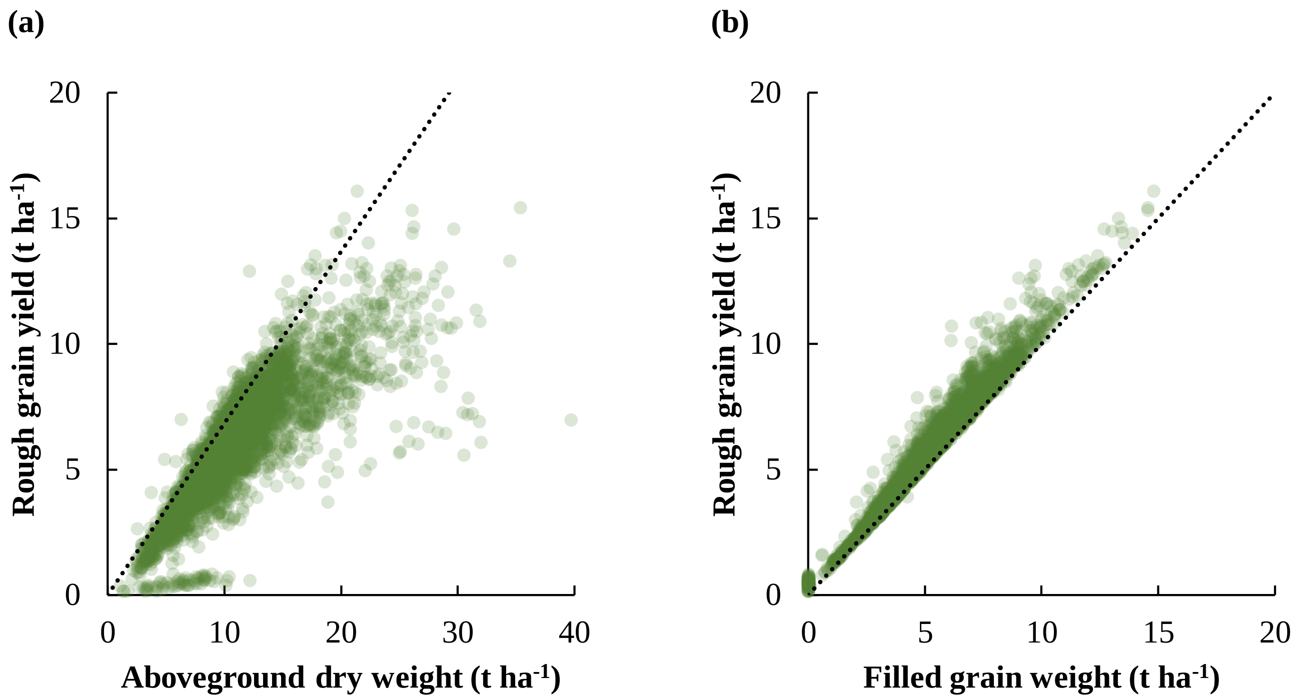

Figure S3. The relationship between the rough grain yield, aboveground dry weight and the filled grain weight.

(a) The relationship between rough grain yield and aboveground biomass. The dotted line represents the harvest index = 0.6. (b) The relationship between rough grainy yield and filled grain yield. The dotted line represents 1:1 relationship.

Learning rate \ Batch size	16	32	64	128
**0.0001**	0.225	0.223	0.225	0.225
**0.0002**	0.226	0.224	0.226	0.225
**0.0005**	0.231	0.227	0.228	0.226
**0.0008**	0.232	0.228	0.227	0.229
**0.001**	0.233	0.229	0.232	0.229

Figure S4. The optimization of the learning rate and batch size for the development of CNN model.

The combination of the various learning rate and batch size was tested with 10 replications. The rRMSE for test data was calculated for all of the conditions and averaged.

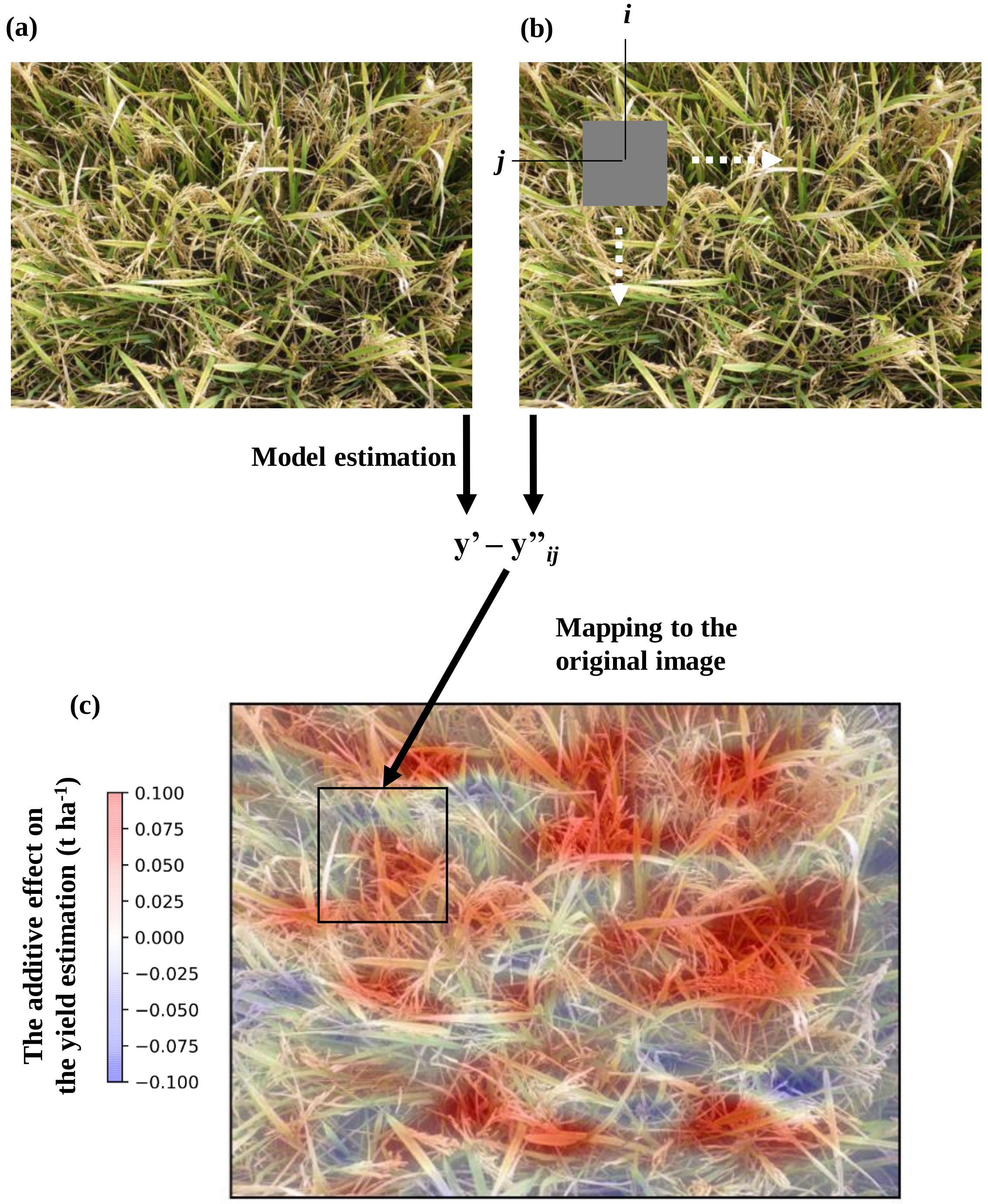

Figure S5. The schematic illustration of the masking analysis to visualize the additive effect of the specific position of the image to the yield estimation.
(a) The original image is input to the model and the estimated value is defined as y’. (b) The specific position (i, j) of the image is masked by gray square sized 30x30 pixels. The masked image is also input to the model and the estimated value is defined as y''_{ij}. (c) The value of $y' - y''_{ij}$ is mapped on the corresponding position of the original image to visualize the additive effect to the yield estimation.

rRMSE (upper triangle); **R^2** (lower triangle)

	20°	30°	40°	50°	60°	70°	80°	90°	Observed
**20°**		0.160	0.237	0.292	0.304	0.321	0.317	0.295	**0.295**
**30°**	0.641		0.179	0.237	0.261	0.278	0.282	0.262	**0.318**
**40°**	0.319	0.540		0.091	0.124	0.143	0.165	0.146	**0.260**
**50°**	0.071	0.240	0.778		0.094	0.105	0.112	0.112	**0.248**
**60°**	0.069	0.166	0.658	0.804		0.076	0.106	0.100	**0.219**
**70°**	0.017	0.091	0.540	0.734	0.871		0.077	0.085	**0.213**
**80°**	0.000	0.036	0.363	0.676	0.756	0.872		0.065	**0.198**
**90°**	0.004	0.062	0.443	0.673	0.807	0.869	0.882		**0.180**
**Obser-ved**	**0.000**	**0.003**	**0.077**	**0.193**	**0.451**	**0.493**	**0.435**	**0.481**	

Figure S6. The effect of the depression angle on the yield estimation.
The matrix of rRMSE and R^2 with the observed and estimated yield for images taken from various depression angles. The depression angle of 90° is identical to the default protocol.

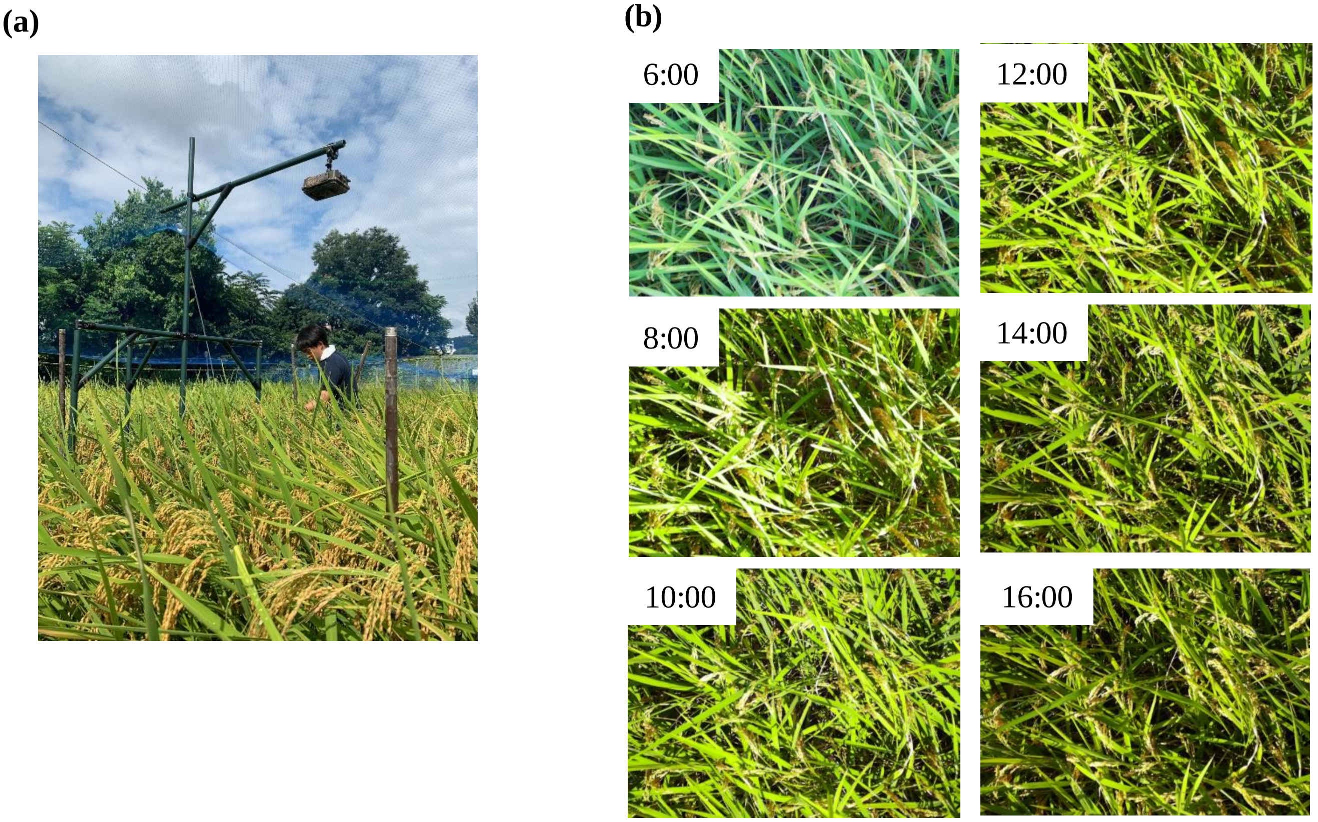

Figure S7. The continuous shooting of the rice canopy image for yield estimation.
(a) Image showing the digital camera fixed above the rice canopy (cv. Koshihikari) .
(b) Images recorded at 0600, 0800, 1000, 1200, 1400, and 1600 hrs on Aug. 29,.

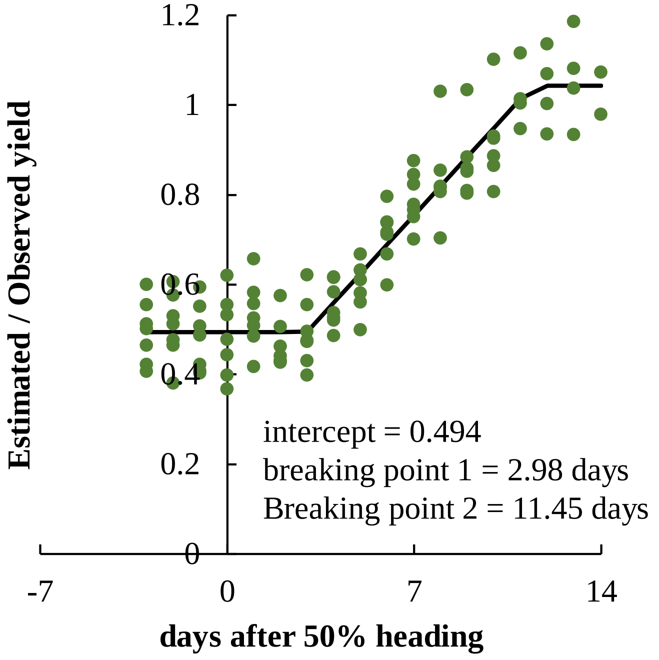

Figure S8. The estimated yield relative to the final yield plotted against days after 50% heading in Madagascar.

The dataset consists of 2 cultivars in 7 harvested plots and the images were taken everyday by fixed camera above the plot. The images was recorded for every 10 min from 1200 (noon) to 1300. The estimated yield is normalized by the final yield observations. The solid line represents the segmented linear regression.
